# Early pregnancy anxiety during the COVID-19 pandemic: preliminary findings from the UCSF ASPIRE study

**DOI:** 10.1186/s12884-022-04595-1

**Published:** 2022-03-31

**Authors:** Jerrine R. Morris, Eleni Jaswa, Amy Kaing, Eduardo Hariton, Miriam Andrusier, Katie Aliaga, Maya Davis, Marcelle I. Cedars, Heather G. Huddleston

**Affiliations:** 1 Department of Obstetrics, Gynecology, & Reproductive Sciences, University of California- San Francisco, 499 Illinois Street Sixth Floor, San Francisco, CA 94158 USA; 2 College of Medicine, SUNY Downstate Health Sciences University, Brooklyn, NY USA 11203; 3 Saint James School of Medicine, Park Ridge, Illinois USA 60068; 4 Department of Medical Oncology, Dana-Farber Cancer Institute, Boston, MA USA 02284

**Keywords:** COVID-19, Pandemic, Anxiety, Pregnancy, Disparities

## Abstract

**Background:**

Antenatal anxiety has been linked to adverse obstetric outcomes, including miscarriage and preterm birth. However, most studies investigating anxiety during pregnancy, particularly during the COVID-19 pandemic, have focused on symptoms during the second and third trimester. This study aims to describe the prevalence of anxiety symptoms early in pregnancy and identify predictors of early pregnancy anxiety during the COVID-19 pandemic.

**Methods:**

We assessed baseline moderate-to-severe anxiety symptoms after enrollment in the UCSF ASPIRE (Assessing the Safety of Pregnancy in the Coronavirus Pandemic) Prospective Cohort from May 2020 through February 2021. Pregnant persons < 10 weeks’ gestation completed questions regarding sociodemographic characteristics, obstetric/medical history, and pandemic-related experiences. Univariate and multivariate hierarchical logistic regression analyses determined predictors of moderate or severe anxiety symptoms (Generalized Anxiety Disorder-7 questionnaire score ≥ 10). All analyses performed with Statistical Analysis Software (SAS®) version 9.4.

**Results:**

A total of 4,303 persons completed the questionnaire. The mean age of this nationwide sample was 33 years of age and 25.7% of participants received care through a fertility clinic. Over twelve percent of pregnant persons reported moderate-to-severe anxiety symptoms. In univariate analysis, less than a college education (*p* < 0.0001), a pre-existing history of anxiety (*p* < 0.0001), and a history of prior miscarriage (*p* = 0.0143) were strong predictors of moderate-to-severe anxiety symptoms. Conversely, having received care at a fertility center was protective (26.6% vs. 25.7%, *p* = 0.0009). COVID-19 related stressors including job loss, reduced work hours during the pandemic, inability to pay rent, very or extreme worry about COVID-19, and perceived stress were strongly predictive of anxiety in pregnancy (*p* < 0.0001). In the hierarchical logistic regression model, pre-existing history of anxiety remained associated with anxiety during pregnancy, while the significance of the effect of education was attenuated.

**Conclusion(s):**

Pre-existing history of anxiety and socioeconomic factors likely exacerbated the impact of pandemic-related stressors on early pregnancy anxiety symptoms during the COVID-19 pandemic. Despite on-going limitations for in-person prenatal care administration, continued emotional health support should remain an important focus for providers, particularly when caring for less privileged pregnant persons or those with a pre-existing history of anxiety.

## Background

The reported prevalence of anxiety during pregnancy varies widely (0.9% to 22.9%), depending on the population studied, with most studies reporting exclusively on symptoms in the second and third trimesters [1–3]. Compared to non-anxious peers, individuals with antenatal anxiety are at increased risk of adverse maternal sequelae including higher rates of negative somatic symptoms, use of sick days during pregnancy, and postpartum depression [4–6]. Pregnancy complications, including miscarriage, preterm birth and low birth weight, have also been linked to maternal anxiety [7, 8], and infants exposed to anxiety in utero exhibit lower developmental scores in infancy [9], behavioral/emotional lability in early childhood [10], and decreased childhood gray matter density [11, 12]. Multiple mechanisms have been proposed to explain the association between maternal anxiety and poor obstetric and childhood outcomes, including stimulated signaling in the hypothalamic–pituitary–adrenal (HPA) axis leading to increased basal cortisol, altered release of proinflammatory markers, and/or a heightened risk for inadequate nutrition or prenatal care [8, 9, 13–15].

The COVID-19 pandemic has resulted in widespread economic and social disruptions, all of which are likely to increase anxiety in susceptible individuals [16, 17]. Those pregnant during this time have faced added uncertainty regarding the potential impact of COVID-19 on pregnancy and limitations in access to in-person prenatal care, both of which may further increase anxiety symptoms [18]. A recent meta-analysis representing 13 studies with over 10,000 pregnant women revealed a 37% prevalence of anxiety during the COVID-19 pandemic, 1.6 times greater than prior to the pandemic [19]. Even after controlling for sociodemographic variables, COVID-19 related stressors were significantly associated with anxiety in pregnancy, underscoring the potential impact of the pandemic on increasing antenatal anxiety and distress [20].

A meta-analysis comprised of several retrospective studies found psychological stress to be a predictor of miscarriage [8]. However, most studies examining anxiety during pregnancy have focused on the later stages of pregnancy and this has persisted since the onset of the COVID-19 pandemic [19, 21–23]. Thus, the prevalence and predictors of anxiety during the first trimester, particularly during a pandemic, remain poorly understood. Given the possible adverse obstetric outcomes associated with antenatal anxiety, this study aims to describe the prevalence of anxiety symptoms early in pregnancy (less than 10 weeks’ gestation) and identify predictors of early pregnancy anxiety during the pandemic.

## Methods

### Data source and sample

This nationwide population-based study includes data from the University of California, San Francisco Assessing the Safety of Pregnancy in the Coronavirus Pandemic Prospective Cohort Study (UCSF ASPIRE). Only pregnant persons < 10 weeks’ gestation were eligible to enroll. Participants were recruited as two distinct samples: 1) a community sample and 2) Society for Assisted Reproductive Technology (SART) sample. The community sample included participants who acquired information about the study through either social media advertisement, the lay press, partnerships with online media companies focused on disseminating information to pregnant persons, traditional clinics providing prenatal care, or word of mouth. The SART sample comprised participants recruited from a network of fertility clinics nationwide with clinical encounters around the time of conception, allowing for precise gestational dating. Both cohorts of ASPIRE participants were prospectively followed throughout their pregnancy and postpartum courses. Prior to enrollment, written, informed consent was obtained. Participants completed baseline questionnaires regarding sociodemographic variables, history of COVID-19 exposure and/or infection, and mental health during the COVID-19 pandemic throughout their pregnancy. Study data were collected and managed using REDCap electronic data capture tools hosted at the University of California, San Francisco [24, 25]. Additionally, participants were sent routine short questionnaires to record their symptoms of COVID-19 infection. Participants were eligible for remuneration after survey completion at multiple time points during this longitudinal study. This study was approved by the UCSF Institutional Review Board.

### Participants

From May 2020 through February 2021, 5,207 pregnant persons across 48 states were enrolled into the UCSF ASPIRE study and completed the baseline questionnaire. Of respondents, 4,303 (82%) completed the Generalized Anxiety Disorder 7 (GAD-7) questionnaire and were included in this analytic sample. The mean age of participants was 33 years of age with over 65% of participants between age 31–40. The regional distribution was similar with the highest recruitment in the West (29.7%) and lowest from the Northeast (19.5%); otherwise, 24.8% and 25.9% were recruited from the Midwest and South, respectively. Close to 26% of participants were recruited from fertility clinics and comprised the SART cohort while roughly 74% were recruited through community.

### Outcomes

Upon enrollment, pregnant persons are asked to complete the GAD-7 questionnaire administered electronically. The GAD-7 is a validated 7-item scale which serves as a screening tool and severity indicator of generalized anxiety disorder and has been recommended to measure perinatal anxiety by the National Institute for Health and Care Excellence [26, 27]. The internal consistency of the GAD-7 has previously been demonstrated to be excellent (Cronbach alpha 0.92) with good test–retest reliability as well (intraclass correlation 0.83) [27]. The questionnaire requires respondents to identify how often they have been bothered by a list of symptoms over the last two weeks including “feeling nervous, anxious, or on edge, not being able to stop or control worrying, and trouble relaxing”, to name a few. The severity of symptoms are categorized as one of four options including “not at all”, “several days”, “more than half the days”, and “nearly every day” which corresponds to 0, 1, 2, or 3 points, respectively. Scoring for anxiety symptom severity is as follows: Minimal (0–4), Mild (5–9), Moderate (10–14), and Severe (15 +). A cut-off of 10 or more indicates clinically significant anxiety symptoms [27].

### Exposures

Characteristics interrogated for their association with anxiety during pregnancy include: maternal age stratified based on the distribution of anxiety in our study (18 to 30, 31 to 35, 36 to 40, 41 or greater years), maternal race/ethnicity (Hispanic, Non-Hispanic White, Black/African American, Asian, Native American, and Pacific Islander), maternal education (Less than a college degree, College degree, Master’s degree, or Professional degree), partner status (Married or partnered, other), income (Less than $99,999, $100,000 to 250,999, $251,000 or more), insurance status (private insurance, other), working outside of the home (none of the time, some of the time, all the time), employment in health care (yes, no), employment status (homemaker, part-time, full-time, unemployed), previous pregnancy and live birth (yes, no), prior miscarriage (yes, no), care through a fertility clinic this pregnancy (yes, no), history of anxiety (yes, no), and history of depression (yes, no).

We also investigated COVID-19 and economic-specific variables, including history of COVID-19 infection among oneself or household contacts (yes, no), COVID-19 death among friends or family residing in the same household (yes, no), job loss (yes, no), reduced hours at work (yes, no), inability to pay rent or mortgage (yes, no), and worry about the health of self or loved ones during the COVID-19 pandemic (yes, no). The Perceived Stress Scale (PSS) [28], a reliable measure of the degree of stress experienced based on situations occurring over the past month, was also investigated to determine the possible impact of COVID-19 related stress on early pregnancy anxiety symptoms. The PSS has an internal reliability of 0.6 and while there are no established cutoffs, we used our population norms to classify low (≤ 4) vs moderate to high (> 4) levels of stress [28].

### Data analysis

This analytic sample was restricted to those who completed the GAD-7 baseline questionnaire. Descriptive characteristics were calculated for the variables of interest. Statistical analyses included univariate logistic regression with determination of predictors of moderate or severe anxiety symptoms (GAD-7: 10 or greater). A multivariate hierarchical logistic regression was performed to calculate adjusted odds ratios (aOR) and 95% confidence intervals (CI) predicting moderate-to-severe anxiety symptoms. Models were adjusted for variables selected as a covariate given its association with anxiety determined a priori (race/ethnicity) or significant in bivariate analyses to a *p* = 0.10 level. Predictors in the first step included demographic characteristics, the second added obstetric and medical history variables, and the third added COVID-19 related characteristics. The third step in the model was implemented to determine how COVID-19 related characteristics contribute additionally to moderate-to-severe anxiety symptoms beyond known risk factors. All analyses were performed with Statistical Analysis Software (SAS®) version 9.4.

## Results

We investigated the association between demographic factors and severity of anxiety symptoms, as estimated by GAD-7 scores. The percent of subjects with no to minimal, mild, moderate, and severe anxiety symptoms was 60.4%, 26.9%, 8.8%, and 3.8%, respectively (Table 1). Rates of moderate-to-severe anxiety symptoms increased with younger age, lower education, unpartnered status, and lack of private insurance prior to pregnancy. There was a trend toward higher income being protective against moderate-to-severe anxiety symptoms as those with a household income of $100–250,999 were less likely to report symptoms compared to those with a household income of less than $100,000; this association was not exhibited among those with a household income of $251,000 or more. Current full-time employment and employment in health care were protective against moderate-to-severe anxiety symptoms (Table 2).

**Table 1:** Sociodemographic and Medical Characteristics of pregnant persons less than 10 weeks’ gestation by anxiety symptoms (*N* = 4,303)

	**Total *N* (%)**	**No or Mild Anxiety**	**Moderate or Severe Anxiety**
**Sociodemographic Characteristics**			
*Participant Race/Ethnicity*	**4,274**	**3,737**	**537**
White, non-Hispanic	3,514 (82.2)	3,090 (82.7)	424 (79.0)
Black/African American	136 (3.2)	113 (3.0)	23 (4.3)
Hispanic/Latinx	404 (9.5)	345 (9.2)	59 (11.0)
Asian	204 (4.8)	177 (4.7)	27 (5.0)
Native American	12 (0.3)	8 (0.2)	4 (0.7)
Pacific Islander	4 (0.1)	4 (0.1)	0 (0.0)
*Participant Age*	**3,789**	**3,319**	**470**
18–30	1,127 (29.7)	950 (28.6)	177 (37.7)
31–35	1,660 (43.8)	1469 (44.3)	191 (40.6)
36–40	826 (21.8)	745 (22.4)	81 (17.2)
41 +	176 (4.7)	155 (4.7)	21 (4.5)
*Household Income*	**4,203**	**3,676**	**527**
< $99,999	1,513 (36.0)	1,230 (33.5)	283 (53.7)
$100,000–250,999	2,098 (49.9)	1,912 (52.0)	186 (35.3)
$251,000 +	592 (14.1)	534 (14.5)	58 (11.0)
*Highest level education completed*	**4,290**	**3,750**	**540**
< College	771 (18.0)	605 (16.1)	166 (30.7)
College	1,413 (32.9)	1,244 (33.2)	169 (31.3)
Master’s	1,396 (32.5)	1,243 (33.2)	153 (28.3)
Professional degree	710 (16.6)	658 (17.6)	52 (9.6)
*Pre-pregnancy insurance*	**4,265**	**3,726**	**539**
Other (including Medi-Cal, Tricare, other)	539 (12.6)	445 (11.9)	114 (21.1)
Private Insurance	3,726 (87.4)	3,281 (88.1)	425 (78.9)
*Partnership status*	**4,280**	**3,741**	**539**
Unpartnered	211 (4.9)	157 (4.2)	54 (10.0)
Married or Partnered	4,069 (95.1)	3,584 (95.8)	485 (90.0)
*Region of residence*	**4,241**	**3,704**	**537**
Northeast	829 (19.6)	724 (19.6)	105 (19.6)
Midwest	1,053 (24.8)	934 (25.2)	119 (22.2)
South	1,099 (25.9)	943 (25.5)	156 (29.1)
West	1,260 (29.7)	1,103 (29.8)	157 (29.2)
*Employment status*	**4,289**	**3,749**	**540**
Full-time	3,168 (73.9)	2,799 (74.7)	369 (68.3)
Other (part-time, homemaker, unemployed)	1,121 (26.1)	950 (25.3)	171 (31.7)
*Employed in health care*	**3,644**	**3,211**	**433**
Yes	1,454 (39.9)	1,310 (40.8)	144 (33.3)
No	2,190 (60.1)	1,901 (59.2)	289 (66.7)
**Obstetric and Medical history**			
*Prior pregnancy*	**4,293**	**3,750**	**543**
Yes	2,713 (63.2)	2,356 (62.8)	357 (65.7)
No	1,580 (36.8)	1,394 (37.2)	186 (34.3)
*Prior live birth*	**2,713**	**2,356**	**357**
Yes	2,055 (75.8)	1,795 (76.2)	260 (72.8)
No	658 (24.2)	561 (23.8)	97 (27.2)
*History of prior miscarriage*	**2,723**	**2,365**	**358**
Yes	1,198 (44.0)	1,019 (43.1)	179 (50.0)
No	1,525 (56.0)	1,346 (56.9)	179 (50.0)
*Receipt of care through fertility clinic this pregnancy*	**4,295**	**3,752**	543
Yes	1,106 (25.7)	998 (26.6)	108 (19.9)
No	3,189 (74.3)	2,754 (73.4)	435 (80.1)
*History of anxiety disorder prior to pregnancy*	**4,303**	**3,759**	**544**
Yes	1,059 (24.6)	784 (20.9)	275 (50.5)
No	3,244 (75.4)	2,975 (79.1)	269 (49.5)
*History of depression prior to pregnancy*	**4,303**	**3,759**	**544**
Yes	898 (20.9)	669 (17.8)	229 (42.1)
No	3,405 (79.1)	3,090 (82.2)	315 (57.9)
**COVID-specific characteristics**			
*History of obtaining a SARS-CoV-2 test this pregnancy*	**4,298**	**3,755**	**543**
Yes	2,558 (59.5)	2,226 (59.3)	332 (61.1)
No	1,740 (40.5)	1,529 (40.7)	211 (38.9)
*History of a positive SARS-CoV-2 test this pregnancy*	**2,534**	**2,204**	**330**
Yes	363 (14.3)	313 (14.2)	50 (15.1)
No	2,171 (85.7)	1,891 (85.8)	280 (84.9)
*Household contact diagnosed with COVID-19 since pregnant*	**4,303**	**3,759**	**544**
Yes	348 (8.1)	306 (8.1)	42 (7.7)
No	3,955 (91.9)	3,453 (91.9)	502 (92.3)
*Household contact deceased from COVID-19 since pregnant*	**4,303**	**3,759**	**544**
Yes	20 (0.5)	11 (0.3)	9 (1.7)
No	4,283 (99.5)	3,748 (99.7)	535 (98.3)
*Job loss since onset of COVID-19 pandemic*	**4,303**	**3,759**	**544**
Yes	358 (8.3)	264 (7.0)	94 (17.3)
No	3,945 (91.7)	3,495 (93.0)	450 (82.7)
*Reduced hours at work since onset of COVID-19 pandemic*	**4,303**	**3,759**	**544**
Yes	552 (12.8)	450 (12.0)	102 (18.7)
No	3,751 (87.2)	3,309 (88.0)	442 (81.3)
*Inability to pay rent or mortgage since onset of COVID-19 pandemic*	**4,303**	**3,759**	**544**
Yes	172 (4.0)	101 (2.7)	71 (13.1)
No	4,131 (96.0)	3,658 (97.3)	473 (86.9)
*Very or Extremely Worried about COVID-19*	**4,294**	**3,752**	**542**
Yes	1,602 (37.3)	1,262 (33.6)	340 (62.7)
No	2,692 (62.7)	2,490 (66.4)	202 (37.3)
*Perceived stress over the past month*	**4,277**	**3,738**	**539**
*Low*	2,262 (52.9)	2,200 (58.9)	62 (11.5)
*Moderate-High*	2,015 (47.1)	1,538 (41.1)	477 (88.5)

**Table 2:** Univariate analysis of variables associated with moderate to severe anxiety symptoms among pregnant persons (*N* = 4,303)

	**OR**^**a**^	**95% CI**^**b**^	**X**^**2**^*** p*-value**
**Demographic Characteristics**			
*Participant Race/Ethnicity*			**0.1153**
White, non-Hispanic	Ref		
Black/African American	1.48	0.94, 2.35	
Hispanic/Latinx	1.25	0.93, 1.67	
Asian	1.11	0.73, 1.69	
Native American	3.64	1.09, 12.15	
Pacific Islander	< 0.001	< 0.001, > 999.999	
*Participant Age*			**0.0006**
18–30	Ref		
31–35	0.70	0.56, 0.87	
36–40	0.58	0.44, 0.77	
41 +	0.73	0.45, 1.18	
*Household Income*			** < 0.0001**
< $99,999	Ref		
$100,000–250,999	0.42	0.35, 0.52	
$251,000 +	0.47	0.35, 0.64	
*Highest level education completed*			** < 0.0001**
Professional degree	Ref		
Master’s	1.56	1.12, 2.16	
College	1.72	1.24, 2.38	
< College	3.47	2.50, 4.83	
*Pre-pregnancy insurance*			** < 0.0001**
Private Insurance	Ref		
Other (including Medi-Cal, Tricare, other)	1.98	1.57, 2.49	
*Partnership status*			** < 0.0001**
Married or Partnered	Ref		
Unpartnered	2.54	1.84, 3.51	
*Employment status*			**0.0018**
Full-time	Ref		
Other (part-time, homemaker, unemployed)	1.37	1.12, 1.66	
*Employed in health care*			**0.0027**
No	1.38	1.12, -1.71	
Yes	Ref		
**Obstetric and Medical history**			
*Previous live birth*			**0.1680**
Yes	Ref		
No	1.19	0.93, 1.54	
*History of prior miscarriage*			**0.0143**
No	Ref		
Yes	1.32	1.06, 1.65	
*Receipt of care through fertility clinic this pregnancy*			**0.0009**
Yes	Ref		
No	1.46	1.17, 1.82	
*History of anxiety disorder prior to pregnancy*			** < 0.0001**
No	Ref		
Yes	3.88	3.22, 4.67	
*History of depression prior to pregnancy*			** < 0.0001**
No	Ref		
Yes	3.36	2.78, 4.06	
**COVID-19 specific characteristics**			
*History of obtaining a SARS-CoV-2 test this pregnancy*			**0.4091**
No	Ref		
Yes	1.08	0.90, 1.30	
*History of a positive SARS-CoV-2 test this pregnancy*			**0.6460**
No	Ref		
Yes	1.08	0.78, 1.49	
*Household contact diagnosed with COVID-19 since pregnant*			**0.7371**
No	Ref		
Yes	0.94	0.68, 1.32	
*Household contact deceased from COVID-19 since pregnant*			**0.0001**
No	Ref		
Yes	5.73	2.36, 13.90	
*Job loss since onset of COVID-19 pandemic*			** < 0.0001**
No	Ref		
Yes	2.77	2.14, 3.57	
*Reduced hours at work since onset of COVID-19 pandemic*			** < 0.0001**
No	Ref		
Yes	1.70	1.34, 2.15	
*Inability to pay rent or mortgage since onset of COVID-19 pandemic*			** < 0.0001**
No	Ref		
Yes	5.44	3.94, 7.47	
*Very or Extremely Worried about COVID-19*			** < 0.0001**
No	Ref		
Yes	3.32	2.76, 4.00	
*Perceived stress over the past month*			** < 0.0001**
*Low*	Ref		
*Moderate-High*	11.00	8.38, 14.45	

^a^*OR* odds ratio

^b^*CI* confidence interval

We investigated the association between reproductive and medical history on anxiety symptoms. Persons with a history of a prior miscarriage were more likely to report greater anxiety symptoms. A history of anxiety and/or depression was associated with increased moderate-to-severe anxiety symptoms. Interestingly, persons who reported receipt of care through a fertility clinic this pregnancy had lower prevalence of moderate-to-severe anxiety symptoms (Table 2).

Finally, experiences and stressors unique to the COVID-19 pandemic were explored as a factor in increased moderate-to-severe anxiety. Pregnant persons reporting exposure to stressors such as job loss, reduced hours at work, or inability to pay rent or mortgage were more likely to report higher anxiety symptom severity. Furthermore, those who reported feeling very or extremely worried about the health of themselves or loved ones during the COVID-19 pandemic or the loss of a friend or family member residing in the same household due to infection with SARS-CoV-2 were more likely to report increased moderate-to-severe anxiety symptoms. Lastly, respondents who reported moderate-to-high perceived stress over the past month were more likely to report moderate-to-severe anxiety symptoms as compared to those with low perceived stress (Table 2).

In the first step of the multivariate hierarchical logistic regression modeling, demographic characteristics associated with moderate-to-severe anxiety determined either a priori or via significance in univariate analyses were considered, including race/ethnicity, age, household income, highest education completed, pre-pregnancy insurance, partnership status, and employment metrics (Table 3). Participants who reported a higher income were less likely to report moderate-to-severe anxiety symptoms. Conversely, women with less than a professional degree, unpartnered, or working in a profession other than health care were more likely to report moderate-to-severe anxiety symptoms.

**Table 3:** Hierarchical Logistic regression predicting moderate to severe anxiety symptoms from demographic, medical, and COVID-19 related variables (*N* = 4,303)

	**Step 1**		**Step 2**		**Step 3**	
	**aOR**^**a**^	**95% CI**^**b**^	**aOR**^**a**^	**95% CI**^**b**^	**aOR**^**a**^	**95% CI**^**b**^
**Demographic Characteristics**						
*Participant Race/Ethnicity*						
White, non-Hispanic	Ref		Ref		Ref	
Black/African American	0.67	0.44, 1.68	0.99	0.45, 2.16	0.98	0.42, 2.29
Hispanic/Latinx	0.95	0.65, 1.40	1.22	0.73, 2.05	1.08	0.62,1.89
Asian	1.14	0.66, 1.97	1.62	0.84, 3.20	1.33	0.63, 2.80
Native American	3.74	0.90, 15.52	3.64	0.63, 21.19	3.14	0.44, 22.51
Pacific Islander	< 0.001	< 0.001, > 999.999	< 0.001	< 0.001, > 999.999	< 0.001	< 0.001, > 999.999
*Participant Age*						
18–30	Ref		Ref		Ref	
31–35	0.90	0.69, 1.17	0.86	0.59, 1.25	0.88	0.58, 1.32
36–40	0.72	0.51, 1.01	0.75	0.48, 1.17	0.73	0.45, 1.18
41 +	0.95	0.55, 1.63	1.07	0.55, 2.06	1.20	0.59, 2.45
* Household Income*						
< $99,999	Ref		Ref		Ref	
$100,000–250,999	0.60***	0.46, 0.79	0.68*	0.47, 0.98	0.79	0.53, 1.18
$251,000 +	0.76	0.51, 1.13	0.85	0.49, 1.44	0.98	0.55, 1.75
*Highest level education completed*						
Professional degree	Ref		Ref		Ref	
Master's	1.62*	1.10, 2.40	1.29	0.79, 2.11	1.24	0.74, 2.08
College	1.46	0.98, 2.18	1.21	0.73, 2.00	1.21	0.71, 2.07
< College	2.58***	1.64, 4.07	2.21**	1.25, 3.89	1.85	1.00, 3.45
*Pre-pregnancy insurance*						
Private Insurance	Ref		Ref		Ref	
Other (including Medi-Cal, Tricare, other)	1.21	0.83, 1.75	1.26	0.79, 2.01	1.11	0.65, 1.88
*Partnership status*						
Married or Partnered	Ref		Ref		Ref	
Unpartnered	1.60*	1.02, 2.52	1.46	0.82, 2.60	1.30	0.69, 2.46
*Employment status*						
Full-time	Ref		Ref		Ref	
Other (part-time, homemaker, unemployed)	0.96	0.69, 1.34	0.88	0.59, 1.32	0.83	0.53, 1.30
*Employed in health care*						
Yes	Ref		Ref		Ref	
No	1.28*	1.01, 1.62	1.22	0.89, 1.66	1.21	0.86, 1.70
Obstetric and Medical history						
*History of prior miscarriage*						
No			Ref		Ref	
Yes			1.27	0.94, 1.72	1.26	0.91, 1.73
*Receipt of care through fertility clinic this pregnancy*						
Yes			Ref		Ref	
No			0.74	0.52, 1.06	0.80	0.55, 1.18
*History of anxiety disorder prior to pregnancy*						
No			Ref		Ref	
Yes			3.16***	2.23, 4.46	2.49***	1.71, 3.62
*History of depression prior to pregnancy*						
No			Ref		Ref	
Yes			1.28	0.89, 1.85	1.17	0.78, 1.74
**COVID-19 specific characteristics**						
*Household contact deceased from COVID-19 since pregnant*						
No					Ref	
Yes					9.40**	**1.75, 50.43**
*Job loss since onset of COVID-19 pandemic*						
No					Ref	
Yes					1.27	0.68, 2.37
*Reduced hours at work since onset of COVID-19 pandemic*						
No					Ref	
Yes					1.10	0.70, 1.73
*Inability to pay rent or mortgage since onset of COVID-19 pandemic*						
No					Ref	
Yes					1.53	0.77, 3.05
*Very or Extremely Worried about COVID-19*						
No					Ref	
Yes					2.43***	**1.77, 3.33**
*Perceived stress over the past month*						
*Low*					Ref	
*Moderate-High*					6.86***	**4.54, 10.36**
	C = 0.637		C = 0.707		C = 0.828	

^a^*aOR* adjusted odds ratio including all variables listed in table

^b^*CI* confidence interval

^*^p < 0.05, **p < 0.01, ***p < 0.001

In the second step of the model, obstetric (history of prior miscarriage and receipt of care through a fertility clinic) and medical variables (history of anxiety or depression) were added (Table 3). A pre-existing history of anxiety conferred a 3.2 times greater likelihood of reporting moderate-to-severe anxiety symptoms even after controlling for demographic characteristics (aOR 3.16, 95% CI [2.23–4.46]). Obtaining less than a college degree remained a significant predictor of moderate-to-severe anxiety even after the adjustment for these factors (aOR 2.205, 95% [1.25–3.89]).

COVID-19 specific characteristics including having a household contact who died from COVID-19, job loss or reduced hours at work, inability to pay rent or mortgage, very or extreme worry about COVID-19, and perceived stress over the past month during the COVID-19 pandemic were entered into the third and final step of the model (Table 3). This addition delineated the magnitude to which the pandemic itself contributed to moderate-to-severe anxiety symptoms within the ASPIRE cohort. While the prevalence was low within this population, having a household contact deceased from COVID-19 during pregnancy increased the report of moderate-to-severe anxiety symptoms by ninefold after adjusting for demographic and medical characteristics (aOR 9.40, 95% CI [1.75–50.43]). Similarly, participants reporting moderate-to-high perceived stress over the past month were 6.9 times more likely to report moderate-to-severe anxiety symptoms (aOR 6.86, 95% CI [4.54–10.36]). As expected, a history of an anxiety disorder prior to pregnancy remained a significant predictor of moderate-to-severe anxiety (aOR 2.49, 95% CI [1.71, 3.62]). Lastly, while less than a college education was no longer significant in the final model, there was a trend toward less than a college education predicting moderate-to-severe anxiety (aOR 1.85, 95% CI [1.00–3.45]).

## Discussion

We sought to determine the prevalence of moderate to severe anxiety symptoms among nationwide pregnant persons at less than 10 weeks’ gestation during the COVID-19 pandemic. Between May 2020 through February 2021, over 4,300 pregnant persons from the ASPIRE prospective cohort responded to a validated measure of anxiety symptoms (GAD-7). We found that 12.6% of pregnant persons reported anxiety in the moderate-to-severe range, and the hierarchical regression showed that in addition to lower maternal education and a pre-existing history of anxiety, COVID-19 specific concerns or experiences were associated with these more severe symptoms.

The lifetime prevalence of reported anxiety disorders is close to 30% with a greater risk exhibited among women as compared to men [29]. Prior to the COVID-19 pandemic, a meta-analysis inclusive of 70 studies reporting the prevalence of self-reported anxiety symptoms across pregnancy was 22.9% [1]. A recent meta-analysis since the onset of the COVID-19 pandemic revealed a pooled prevalence of anxiety symptoms among pregnant women of 34% (95% CI: 22–47%) [19, 30]. In both meta-analyses, different countries and metrics for anxiety symptoms were included making comparisons with the current study challenging, with only one study included that investigated U.S women using the GAD-7 since the onset of the COVID-19 pandemic. In this study, Preis et al. (2021) used social media to recruit pregnant women between April–May 2020 for an internet-based study of 1,367 participants (mean gestational age = 34 weeks at enrollment) and found that 35% of respondents reported moderate-to-severe anxiety symptoms [21]. In contrast, Liu et al. (2020) surveyed 1,061 pregnant and recently postpartum U.S. women also identified through social media regarding anxiety symptoms using the GAD-7. The prevalence of moderate-to-severe anxiety symptoms in this population was closer to 22% [22]. We postulate the lower rates of anxiety symptoms found in our study may be linked to demographic differences between cohorts, extended recruitment possibly encompassing participants with varied perceived COVID-19 risks, and/or the earlier gestational age of our participants as no study to date has focused on exploring the prevalence of anxiety symptoms during the COVID-19 pandemic beginning in the first trimester.

Lower education attainment and a pre-existing history of anxiety have previously been recognized as predictors of moderate-to-severe anxiety symptoms prior to the COVID-19 pandemic [14]. Similar to findings by Moyer et al. (2020), these baseline sociodemographic and medical characteristics remained significant predictors of anxiety during the COVID-19 pandemic [20]. While older age has been shown to be protective against increased anxiety symptoms in some studies, our cohort, restricted to persons of reproductive age, did not reflect this, consistent with findings from other studies exploring maternal anxiety during the COVID-19 pandemic [20, 22, 31].

Respondents who reported being very or extremely worried about personal or a close contact becoming infected with SARS-CoV-2 or losing a loved one during the COVID-19 pandemic were more likely to report more severe symptoms of anxiety even after accounting for preexisting sociodemographic and medical characteristics. These findings were similar to other reports showing the profound contribution of COVID-19 related concerns and experiences on developing anxiety related symptoms [20, 22, 31]. Furthermore, our unique focus on the first trimester underscored the contribution of pandemic-related stressors to anxiety in early pregnancy likely exacerbated in high-risk populations.

The American Congress of Obstetricians and Gynecologists (ACOG) recently emphasized the importance of screening for mood disorders, including anxiety, given increasing awareness of the adverse clinical repercussions of untreated mood disorders in pregnancy [32]. The pandemic has brought about multiple stressors with the potential to further heighten anxiety for pregnant persons, while at the same time limiting in-person interactions with care providers. The implications of the widespread use of virtual care for pregnancy is unknown. However, in a nationwide survey, less than half of respondents felt they received the same amount or more information compared to a traditional visit [33]. There was a trend towards receipt of care during the first trimester through a fertility clinic being protective against moderate-to-severe anxiety symptoms in pregnancy. These patients are often seen routinely throughout the first trimester which has largely continued throughout the pandemic; this may have contributed to decreased anxiety symptoms in this population. Furthermore, the results of our study highlight the importance of accommodating mental health screening in virtual visits and suggests the importance of allowing for particular focus on those with lower educational attainment and high levels of concern regarding COVID-19.

### Strengths and limitations

An important strength of this work is the large cohort size and its national representation. Beyond the pandemic, this study represents one of the few studies to date to capture the prevalence and risk factors for anxiety symptoms during the first trimester. These results therefore represent novel information regarding a gestational time that has been historically difficult to access. We anticipate that future results from this longitudinal cohort will clarify the natural history and later pregnancy implications of anxiety initially identified in the first trimester. Nevertheless, there are several limitations to be considered as well. Although we utilized a gold-standard instrument for interrogating anxiety symptoms, questionnaire-based assessments lack the rigor of a clinical interview. Further, our cohort represents a population of persons interested and able to participate in a longitudinal seroprevalence study of COVID-19, and our recruitment scheme utilized both social media and fertility clinic-based recruitment and required enrollment prior to 10 weeks of gestation. As such, while this study is nationwide, it includes predominantly White, non-Hispanic women of higher socioeconomic status, thus, the generalizability of our findings should be considered with these factors in mind. We did not see a difference in anxiety symptoms based on the month enrolled into the cohort. However, as burden from the COVID-19 has differed by region throughout this period, it is possible there is an association between regional surge and anxiety symptoms we were unable to disentangle. While this could be a potential factor associated with the presence of anxiety symptoms, we do not feel this confounded the associations otherwise seen in this analysis. We sought to ascertain if working on-site differentially impacted anxiety symptoms when compared to employment that allowed some or completely virtual options. While this variable was not a significant predictor in our analysis, we acknowledge the structure of the question may have impacted any potential association. Finally, our findings likely do not accurately reflect the experience of persons with less interest in or capacity for research participation or the experience of those who did not become aware of their pregnancy until after 10 weeks.

## Conclusions

A prior history of anxiety, low maternal educational attainment and concerns specific to the COVID-19 pandemic were significant predictors of moderate-to-severe anxiety in the ASPIRE cohort. Continued emotional health support should remain an important focus for providers, particularly when caring for those who are underserved, have a preexisting history of anxiety, and early in pregnancy when care may be limited. With limitations in in-person prenatal care as a function of the COVID-19 pandemic, continued attention to mental health screening and appropriate intervention should remain an important focus for providers. Furthermore, future studies are essential to explore the continued impact of the pandemic on antenatal and postnatal anxiety symptoms especially among the most vulnerable populations.

## Data Availability

The datasets generated and/or analyzed during the current study are not publicly available since the study is still actively collecting data but are available from the corresponding author on reasonable request.

## Abbreviations

HPA: Hypothalamic–pituitary–adrenal
SART: Society for Reproductive Technology
GAD-7: Generalized Anxiety Disorder-7
aOR: Adjusted odds ratio
SAS: Statistical Analysis Software
ACOG: American Congress of Obstetricians and Gynecologists
